# The clinicopathological characteristics of gastric cancer and precancerous conditions in gastric DLBCL and MALT lymphoma patients: a multi-center retrospective study

**DOI:** 10.1080/07853890.2023.2193423

**Published:** 2023-05-15

**Authors:** Yun Feng, Tian-Jiao Duan, Qing Huang, Zhi-Yi Li, Ya-Ping Liu, Miao-Sha Luo, Gui-Fang Lu, Wen Shi, Zhi-Yong Zhang, Hong-Xia Li

**Affiliations:** a The First Affiliated Hospital of Xi’an Jiao Tong University, Xi’an, People’s Republic of China; b Department of Gastroenterology, Shaanxi Provincial People’s Hospital, Xi’an, People’s Republic of China; c Department of Gastroenterology, Beijing Tsinghua Changgung Hospital, School of Clinical Medicine, Tsinghua University, Beijing, People’s Republic of China; d Department of Hematology, The First Affiliated Hospital of Xi’an Jiao Tong University, Xi’an, People’s Republic of China

**Keywords:** Primary gastric lymphoma, mucosa-vassociated lymphoid tissue lymphoma, diffuse large B-cell lymphoma, gastric cancer, precancerous conditions, *Helicobacter pylori*

## Abstract

**Objective:**

The objective of this study is to explore the clinicopathological characteristics of gastric cancer and precancerous conditions in patients with primary gastric lymphoma.

**Methods:**

We analyzed 474 cases of primary gastric lymphoma, mainly DLBCL and MALT, from three clinical centres retrospectively, and compared the clinicopathological parameters of primary gastric lymphoma patients complicated with gastric cancer, precancerous conditions, or with no complications.

**Results:**

A total of 5.1% of the patients with primary gastric lymphoma were diagnosed with gastric cancer, including metachronous gastric adenocarcinoma (3.2%) and synchronous gastric adenocarcinoma (1.9%). Of the patients with gastric lymphoma, 14.6% had precancerous conditions including atrophy (14.6%), intestinal metaplasia (8.9%), and low-grade intraepithelial neoplasia (1.9%). Primary gastric lymphoma patients with an ulcerative type (*p* = 0.009) and Lugano classification stage IIE + IV (*p* < 0.001) lymphoma had a higher risk of complicating with gastric cancers or precancerous conditions. The rate of infection of *Helicobacter pylori* (Hp) was 68.4% in patients with primary gastric lymphoma, which was higher in patients with MALT lymphoma (*p* < 0.001), Lugano classification stage I + II (*p* < 0.001), and patients complicated with precancerous conditions and gastric cancer (*p* < 0.001), especially gastric cancer of the intestinal type (*p* = 0.04). Gastric cancer (95.8%) and precancerous conditions (91.3%) occurred mostly in Hp-infected primary gastric lymphoma patients, with a minor subset of Hp-eradicated patients. Primary gastric lymphoma patients had a higher detection rate of early gastric cancer (25.0%) and a five-year survival rate (40.0%) than the general Chinese population.

**Conclusions:**

Patients with primary gastric lymphoma have a high risk of developing gastric cancer and precancerous conditions, and this risk may be related to *Helicobacter pylori* infection. Follow-up of primary gastric lymphoma provides an opportunity for the detection of early gastric cancer.

**Key messages**
- 5.1% of the patients with primary gastric lymphoma were diagnosed with gastric cancer.
- 14.6% of the patients with gastric lymphoma had premalignant lesions including atrophy (14.6%), intestinal metaplasia (8.9%), and low-grade intraepithelial neoplasia (1.9%).
- Primary gastric lymphoma patients complicating with gastric cancer had a higher infection rate of *Helicobacter pylori* (100.0%), a detection rate of early gastric cancer (25.0%) and a five-year survival rate (40.0%) than the general Chinese population.

## 1. Introduction

The incidence and mortality of gastric cancer rank first among malignant tumours of the stomach, with about 1.09 million new cases and 768,000 deaths each year globally [1]. Approximately 90% of gastric cancer is gastric adenocarcinoma [2], and the next primary gastric lymphoma is the second most common gastric malignant tumour [3]. In treating these cancers, clinicians need to differentiate between gastric cancer and primary gastric lymphoma if masses, ulcers, or other lesions are found in the stomach, and current identification methods include pathology biopsy and endoscopy, as well as other imaging examinations [4]. However, rare cases of primary gastric lymphoma complicated with synchronous gastric cancer and metachronous gastric cancer have attracted the attention of clinical researchers in particular. The occurrence of primary gastric lymphoma is closely related to *Helicobacter pylori* (*H. pylori*), especially mucosa-associated lymphoid tissue (MALT) lymphomas and some diffuse large B-cell lymphomas (DLBCL) [5]. *H. pylori* infection can lead to chronic inflammation, atrophy, intestinal metaplasia, low-grade intraepithelial neoplasia, and the intestinal type of gastric adenocarcinoma [6–8]. However, few studies have shown that patients with primary gastric lymphoma have a significantly increased risk of developing malignant solid tumours, such as gastric cancer [9,10], and the question of whether patients with primary gastric lymphoma have an increased risk for gastric cancer and precancerous conditions remains controversial [11,12].

This study retrospectively analyzes the clinicopathological characteristics of gastric cancer and precancerous conditions in patients with primary gastric lymphoma from three clinical cancer centres in China and aims to provide clinical evidence for the correlation between primary gastric lymphoma and gastric cancer.

## 2. Materials and methods

### 2.1. Study objects and groups

Our study sample consisted of 474 primary gastric lymphoma patients diagnosed at three Chinese cancer centers between January 2010 to August 2020, including 268 patients from the First Affiliated Hospital of Xi’an Jiao Tong University, 119 patients from Shaanxi Provincial People’ss Hospital, and 87 patients from Beijing Tsinghua Changgung Hospital. Our inclusion criteria were the presence of the following: clinical and pathological diagnosis of primary gastric lymphoma and regular endoscopic follow-up, and our exclusion criteria were the presence of complications with malignant tumours other than the stomach, previous upper digestive tract surgery, failure to cooperate with gastroscopy, serious cardiopulmonary disease, and serious liver, kidney, or mental disease. A diagnosis of primary gastric lymphoma was made based on the Dawson criteria [13]. We broke down the sample into two experimental groups and a control group. First, the gastric cancer group included cases diagnosed with synchronous gastric adenocarcinoma within 6 months or metachronous gastric adenocarcinoma beyond 6 months from primary gastric lymphoma diagnosis. Second, the gastric precancerous conditions group included cases with atrophy, intestinal metaplasia, or low-grade intraepithelial neoplasia at initial endoscopy and during follow-up, excluding patients who progressed to gastric cancer. The cases that didn’t develop gastric cancer or precancerous conditions were placed in the control group.

### 2.2. Gastroscopy

Gastroscopy follow-up of patients diagnosed with primary gastric lymphoma was carried out regularly in accordance with the guidelines presented in Matysiak-Budnik et al. [14]. A standard gastroscopy requires adequate preparation before the examination, where the endoscopist should carefully observe a patient’s *H. pylori* infection status, background mucosa, changes in the original lesions, and suspicious malignant lesions by white light endoscopy, chromoendoscopy, linked colour imaging, narrow band imaging, and magnifying endoscopy. After careful observation, we took a biopsy of suspicious lesions for histopathological examination as well.

### 2.3. Diagnosis of gastric cancer and precancerous conditions

Atrophy and intestinal metaplasia can be diagnosed by gastroscopy or pathology alone. Diagnosis of gastric cancer and low-grade intraepithelial neoplasia must be confirmed by histopathological examination of a biopsy or surgical specimens. Endoscopic manifestations of atrophic gastritis include the pale appearance of gastric mucosa, thinning of the gastric mucosa, increased visibility of vasculature, and loss of gastric folds [15,16]. The degree of atrophic gastritis is assessed using the Kimura-Takemoto classification system or Operative Link for Gastritis Assessment (OLGA) classification [15–17]. The pathological diagnosis of atrophic gastritis is based on the loss of normal glandular epithelium gastric glands [15,16]. Intestinal metaplasia is manifested as a gray-white flat bulge on white light endoscopy, and regular vessels with ridge/tubular or tubulovillous glands, particularly with a light blue crest on magnified narrow band imaging, mostly on an atrophic background [18]. The degree of intestinal metaplasia is assessed using the Operative Link on Gastric Intestinal Metaplasia Assessment (OLGIM) system [15,16]. The pathological manifestations of intestinal metaplasia are divided into complete intestinal metaplasia and incomplete intestinal metaplasia. Complete intestinal metaplasia resembles the small intestinal epithelium, while incomplete intestinal metaplasia resembles the colonic epithelium [19]. The classification of gastric cancer and intraepithelial neoplasia was defined by World Health Organization [20]. Low-grade intraepithelial neoplasia has limited architectural abnormalities and only mild to moderate cytological atypia, with hyperchromatic, elongated, pseudostratified nuclei [21].

### 2.4. *H. pylori* detection and eradication

*H. pylori* infection status was determined by *H. pylori* detection and patients’ histories of eradication therapy. For detection, we used a ^13^C-urea breathing test, a ^14^C-urea breath test, or a rapid urease test to detect *H. pylori*. A positive result for any of the above tests confirmed the presence of an infection with *H. pylori*, which we denote as ‘Hp-infected’, and negative results without previous *H. pylori* eradication therapy were judged to be ‘Hp-uninfected’. Negative results after *H. pylori* eradication therapy was denoted as ‘Hp-eradicated’. In order to avoid false negative test results, we required an interval of at least 4 weeks from antibiotics and at least 2 weeks from proton pump inhibitor and bismuth before *H. pylori* detection could take place. Patients were also required to fast for at least 2 h before the examination.

### 2.5. Clinicopathological data collection

Clinicopathological and demographic data of all the study subjects were also collected and analyzed. Demographic data included age and sex, and the clinicopathological characteristics of primary gastric lymphoma included diagnosis time, pathological type, lesion location, endoscopy manifestation, Lugano Classification, B symptoms, *H. pylori* infection status, treatment and prognosis. The clinicopathological characteristics of gastric cancer included symptoms, diagnosis time, lesion location, largest tumour size, TNM staging, pathological type, Lauren classification, *H. pylori* infection status, treatment and prognosis. Finally, the clinicopathological characteristics of gastric precancerous lesions included diagnosis time, lesion location, pathological type, *H. pylori* infection status, treatment and prognosis as well. We successfully contacted 391 patients by telephone on 22 May 2022 to 124 months (median: 49 months) after their initial diagnosis of primary gastric lymphoma. The remaining patients were lost.

### 2.6. Statistical analysis

We used SPSS 24.0 (SPSS Inc., Chicago, IL, USA) for the statistical analysis of all of our data. Categorical variables were expressed as the number of cases (percentage) and analyzed by Pearson χ^2^ tests or Fisher’s exact test between groups, and continuous variables that conformed to normal distribution were represented by mean ± standard deviation and analyzed by Student’s *t*-tests and ANOVA tests. Continuous variables that didn’t conform to normal distribution were presented as median (interquartile range) and analyzed by Mann–Whitney *U*-tests. The overall survival rate and its risk factors were evaluated by the Kaplan–Meier survival analysis. Our threshold for a statistically significant test result was *p* < 0.05 for all tests.

## 3. Results

### 3.1. Demographic and clinicopathological characteristics of patients with primary gastric lymphoma

**Figure 1.**
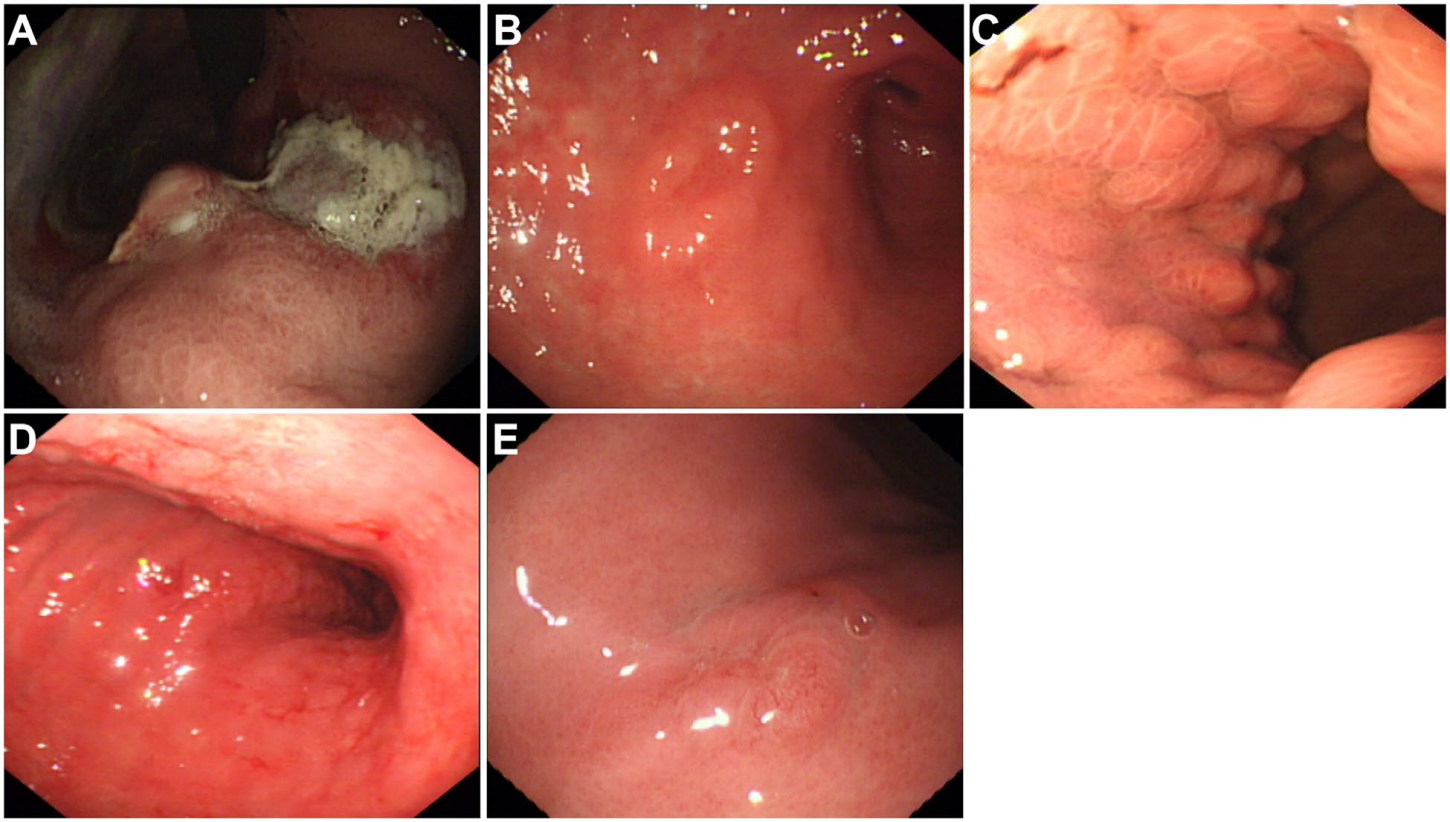
Endoscopic manifestation of primary gastric lymphoma. (A) ulcerative type; (B) mass or polypoid type; (C) fold change type; (D) infiltrative type; (E) superficial type.

In total 474 patients with primary gastric lymphoma were enrolled in our study. As shown in Table 1, the patients’ average age was 52.6 ± 5.5 years (27 to 73 years), and 63.5% of the patients were male. The most common involved locations were the gastric body (55.9%) and gastric antrum (45.1%), and ulcerative type (32.5%) was the most common endoscopic manifestation of primary gastric lymphoma, and the pathological types of primary gastric lymphoma were mainly DLBCL (47.5%) and MALT lymphoma (45.6%). Other pathological types of primary gastric lymphoma included follicular lymphoma (1.9%), mantle cell lymphoma (0.8%), Burkitt lymphoma (0.8%), and T cell lymphoma (3.4%). Most primary gastric lymphomas were assessed as Lugano Classification stage I (43.5%) and stage II (33.1%) without B symptoms (70.9%). Only 9.1% of the patients were Lugano Classification stage IV with bone marrow invasion (5.5%) or supradiaphragmatic nodal involvement (3.6%). Figure 1 shows endoscopic manifestations of primary gastric lymphomas.

**Table 1.** Demographic and clinicopathological characteristics of patients with primary gastric lymphoma, mean ± SD or *N*(%).

Characteristic	Primary gastric lymphoma (*n* = 474)
Sex	
Male	301 (63.5%)
Female	173 (36.5%)
Age, y	52.6 ± 5.5
Lesion location	
Gastric antrum	214 (45.1%)
Gastric body	265 (55.9%)
Gastric fundus	115 (24.3%)
Pylorus	103 (21.7%)
Cardia	42 (8.9%)
Lesion distribution	
Multi-site	78 (16.5%)
Single-site	396 (83.5%)
Endoscopic manifestation	
Ulcerative type	154 (32.5%)
Mass or polypoid type	121 (25.5%)
Fold change type	103 (21.7%)
Infiltrative type	48 (10.1%)
Superficial type	48 (10.1%)
Pathological type	
DLBCL	225 (47.5%)
MALT lymphoma	216 (45.6%)
Others	33 (7.0%)
Lugano classification	
I	206 (43.5%)
II	157 (33.1%)
IIE	68 (14.3%)
IV	43 (9.1%)
B symptoms	
Yes	138 (29.1%)
No	336 (70.9%)

DLBCL: diffuse large B-cell lymphoma; MALT: mucosa-associated lymphoid tissue.

### 3.2. The risk of gastric cancer in patients with primary gastric lymphoma

A total of 5.1% (24/474) of primary gastric lymphoma patients had been diagnosed with gastric cancer, all of which were gastric adenocarcinomas. As shown in Table 2, MALT lymphoma (50%, 12/24) and DLBCL (50%, 12/24) were the main pathological types. Primary gastric lymphoma patients with an ulcerative type (with gastric cancer, 14/24, 58.3% vs. without gastric cancer, 146/450, 32.4%; *p* = 0.009) and Lugano classification stage IIE + IV (with gastric cancer, 14/24, 58.3% vs. without gastric cancer, 113/450, 25.1%; *p* < 0.001) had a higher risk of gastric cancer. The odds ratios for ulcerative type and Lugano classification stage IIE + IV were 2.748 (95% CI 1.248–6.047) and 3.825 (95% CI 1.744–8.392), respectively.

**Table 2.** Demographic and clinicopathological characteristics of primary gastric lymphoma patients with or without gastric cancer, mean ± SD or *N*(%).

Characteristic	With gastric cancer (*n* = 24)	Without gastric cancer (*n* = 450)	*p* Value
Sex			0.741
Male	16 (66.7%)	285 (63.3%)	
Female	8 (33.3%)	165 (36.7%)	
Diagnosis age of lymphoma, y	54.0 ± 8.4	52.2 ± 5.2	0.053
Lesion location of lymphoma			
Gastric antrum	10 (41.6%)	218 (48.4%)	0.517
Gastric body	14 (58.3%)	261 (58.0%)	0.974
Others	4 (16.7%)	54 (12.0%)	0.497
Lesion distribution of lymphoma			
Multi-site	3 (12.5%)	62 (13.8%)	0.859
Single-site	21 (87.5%)	388 (86.2%)	
Endoscopic manifestation of lymphoma			0.009
Ulcerative type	14 (58.3%)	146 (32.4%)	
Other types	10 (41.7%)	304 (67.6%)	
Pathological type of lymphoma			0.485
DLBCL	12 (50.0%)	221 (49.1%)	
MALT lymphoma	12 (50.0%)	204 (45.3%)	
Others	0 (0.0%)	25 (5.6%)	
Lugano classification of lymphoma			< 0.001
I + II	10 (41.6%)	337 (74.9%)	
IIE + IV	14 (58.3%)	113 (25.1%)	
B symptoms of lymphoma			0.807
Yes	7 (29.2%)	121 (26.9%)	
No	17 (70.8%)	329 (73.1%)	

DLBCL: diffuse large B-cell lymphoma; MALT: mucosa-associated lymphoid tissue.

Among the patients with gastric adenocarcinoma, 1.9% (9/474) and 3.2% (15/474) had synchronous gastric cancer and metachronous gastric cancer, respectively. Only one patient who had synchronous gastric cancer was diagnosed at the same time with gastric lymphoma. The median diagnosis interval between metachronous gastric cancer and primary gastric lymphoma was about 3 years (–5 to 11 years). Only one patient who had metachronous gastric cancer was diagnosed before gastric lymphoma. We found no significant differences in sex, age, lesion location, largest tumour size, pathological type, Lauren classification, or TNM stage between the synchronous gastric adenocarcinoma group and the metachronous gastric adenocarcinoma group (Table 3).

**Table 3.** Demographic and clinicopathological characteristics of gastric cancer patients with primary gastric lymphoma, mean ± SD, median (interquartile range) or *N*(%).

Characteristic	Gastric cancer (*n* = 24)		
	Synchronous gastric adenocarcinoma (*n* = 9)	Metachronous gastric adenocarcinoma (*n* = 15)	*p* Value
Sex			>0.999
Male	6 (66.7%)	9 (60.0%)	
Female	3 (33.3%)	6 (40.0%)	
Age, y^a^	54 (43.5)	55 (54)	0.857
Diagnosis interval, y^b^	0 (0)	3 (2)	<0.001
Lesion location of the cancer			>0.999
Gastric antrum	5 (55.6%)	7 (46.7%)	
Gastric body	2 (22.2%)	4 (26.7%)	
Others	2 (22.2%)	4 (26.7%)	
Largest tumor size, cm	4 (3.25)	4 (3)	0.627
Pathological type of cancer			>0.999
Differentiated adenocarcinoma	7 (77.8%)	12 (80.0%)	
Undifferentiated adenocarcinoma	2 (22.2%)	3 (20.0%)	
Lauren classification of cancer			0.829
Intestinal type	6 (66.7%)	8 (53.3%)	
Diffuse type	3 (33.3%)	5 (33.3%)	
Mixed type	0 (0.0%)	2 (13.3%)	
T stage			>0.999
T1	3 (33.3%)	6 (40.0%)	
T2 + T3 + T4	6 (66.7%)	9 (60.0%)	
N stage			>0.999
N0	5 (55.6%)	8 (53.3%)	
N1 + N2 + N3	4 (44.4%)	7 (46.7%)	
M stage			0.326
M0	6 (66.7%)	13 (86.7%)	
M1	3 (33.3%)	2 (13.3%)	
TNM stage			>0.999
Advanced gastric cancer	7 (77.8%)	11 (73.3%)	
Early gastric cancer	2 (22.2%)	4 (26.7%)	

^a^Age of patients when diagnosed with gastric cancer.

^b^Diagnosis interval between gastric cancer and primary gastric lymphoma of each case is presented as an estimated whole year. The diagnosis of gastric cancer earlier than gastric lymphoma is indicated by a negative number, and the diagnosis of gastric cancer later than gastric lymphoma is indicated by a positive number.

**Figure 2.**
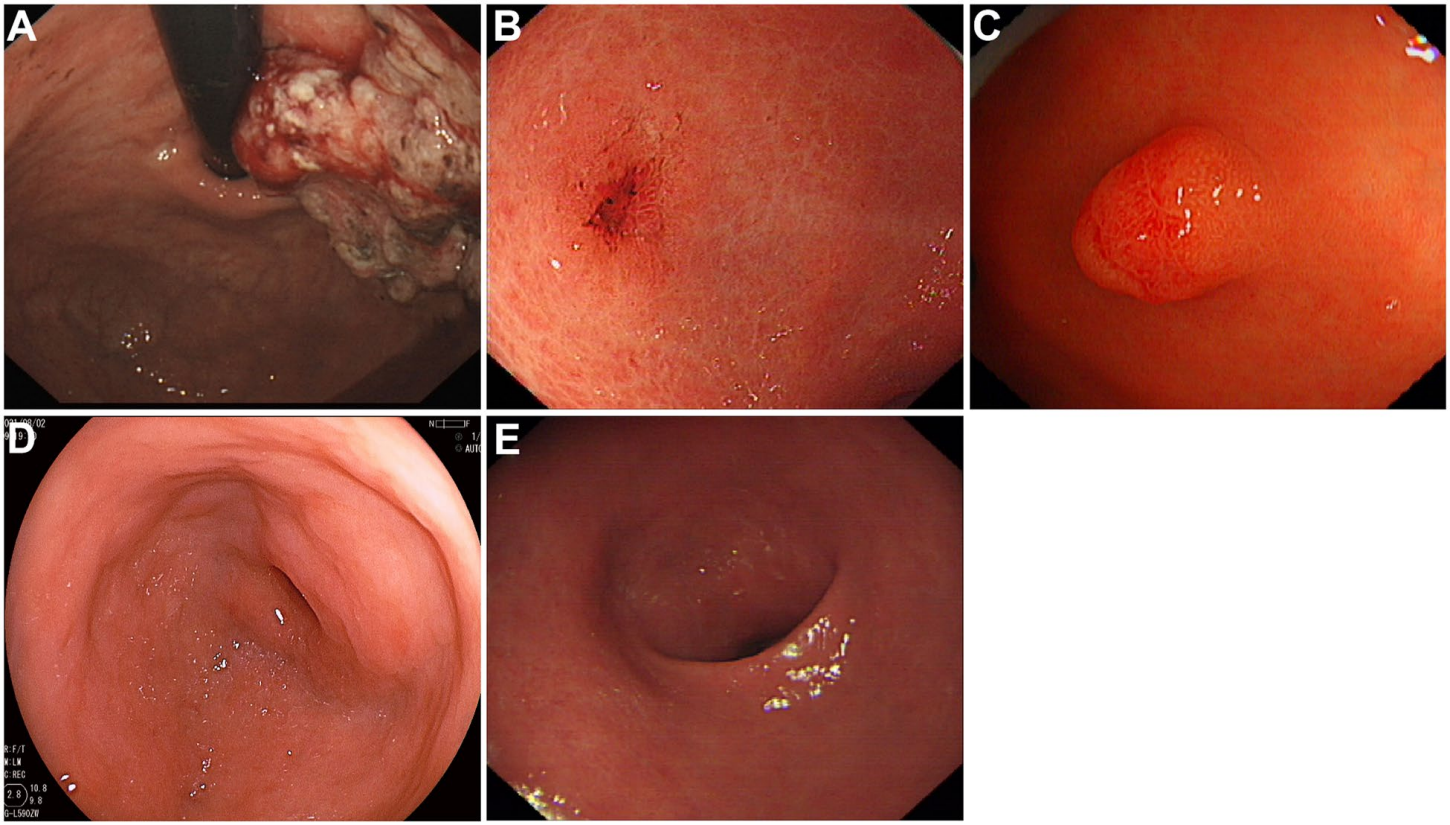
Endoscopic manifestation of gastric cancer and precancerous conditions in primary gastric lymphoma patients. (A) advanced gastric adenocarcinoma; (B) early gastric adenocarcinoma; (C) low-grade intraepithelial neoplasia; (D) intestinal metaplasia; (E) atrophy.

Most primary gastric lymphoma patients who had gastric cancer had advanced gastric cancer (18/24, 75.0%), accounting for 77.8% (7/9) of synchronous gastric adenocarcinoma and 86.7% (11/15) of metachronous gastric adenocarcinoma. Figure 2(A,B) show advanced gastric adenocarcinoma and early gastric adenocarcinoma developed in primary gastric lymphoma patients. The patients with advanced gastric cancer had larger tumour sizes (advanced gastric cancer, 4.8 cm (1.3 cm) vs. early gastric cancer, 3.0 cm (0.9 cm); *p* = 0.001), higher T stage (advanced gastric cancer, T1, 3/18, 16.7% vs. early gastric cancer, T1, 6/6, 100.0%; *p<*0.001), and higher N stage (advanced gastric cancer, N0, 11/18, 61.1% vs. early gastric cancer, N0, 0/6, 0.0%; *p* = 0.009) than those with early gastric cancer.

### 3.3. The risk of gastric precancerous conditions in patients with primary gastric lymphoma

A total of 14.6% (69/474) of the patients with primary gastric lymphoma had gastric precancerous conditions, including atrophy (14.6%, 69/474), intestinal metaplasia (8.9%, 42/474), and low-grade intraepithelial neoplasia (1.9%, 12/474) (Figure 2(C–E)). Endoscopic manifestation (*p* < 0.001) and Lugano classification (*p* < 0.001) of primary gastric lymphoma differed among the groups of patients with gastric cancer, precancerous conditions or not (Table 4). However, more ulcerative-type primary gastric lymphoma was seen in patients with gastric precancerous conditions (gastric precancerous conditions, 38/69, 55.1% vs. control, 108/381, 28.3%; *p* < 0.001) as well as gastric cancer (gastric cancer, 14/24, 58.3% vs. control, 108/381, 28.3%; *p* = 0.004). Primary gastric lymphoma patients with Lugano classification stage IIE + IV developed more gastric precancerous conditions (gastric precancerous conditions, 38/69, 55.1% vs. control, 75/381, 19.7%; *p* < 0.001) and gastric cancer as well (gastric cancer, 14/24, 58.3% vs. control, 75/381, 19.7%; *p* < 0.001). The median diagnosis interval between gastric precancerous conditions and primary gastric lymphoma was about 3 years (3 to 11 years).

**Table 4.** Demographic and clinicopathological characteristics of primary gastric lymphoma patients with gastric cancer and gastric precancerous conditions, mean ± SD, median (interquartile range), or *N*(%).

Characteristic	Gastric cancer (*n* = 24)	Gastric precancerous conditions (*n* = 69)	Control (*n* = 381)	*p* Value
Sex				0.804
Male	16 (66.7%)	46 (66.7%)	240 (63.0%)	
Female	8 (33.3%)	23 (33.3%)	141 (37.0%)	
Diagnosis age of lymphoma, y	52 (5.5)	51 (12)	53 (9)	0.502
Diagnosis age of gastric cancer or precancerous conditions, y	55 (5)	55 (9)	/	0.795
Diagnosis interval^a^, y	2 (5)	3 (3)	/	0.102
Lesion location of lymphoma				
Gastric antrum	10 (41.6%)	34 (49.3%)	184 (48.3%)	0.802
Gastric body	14 (58.3%)	32 (46.4%)	229 (60.1%)	0.104
Others	4 (16.7%)	7 (10.1%)	47 (12.3%)	0.697
Endoscopic manifestation of lymphoma				
Ulcerative type	14 (58.3%)	38 (55.1%)	108 (28.3%)	< 0.001
Mass or polypoid type	5 (20.8%)	15 (21.7%)	77 (20.2%)	
Other type	5 (20.8%)	16 (23.2%)	196 (51.4%)	
Pathological type of lymphoma				0.816
DLBCL	12 (50.0%)	35 (50.7%)	186 (48.8%)	
MALT lymphoma	12 (50.0%)	30 (43.5%)	174 (45.7%)	
Others	0 (0.0%)	4 (5.8%)	21 (5.5%)	
Lugano classification of lymphoma				< 0.001
I + II	10 (41.6%)	31 (44.9%)	306 (80.3%)	
IIE + IV	14 (58.3%)	38 (55.1%)	75 (19.7%)	
B symptoms of lymphoma				0.268
Yes	7 (29.2%)	24 (34.8%)	97 (25.5%)	
No	17 (70.8%)	45 (65.2%)	284 (74.5%)	

DLBCL: diffuse large B-cell lymphoma; MALT: mucosa-associated lymphoid tissue.

^a^Diagnosis interval between gastric cancer or gastric precancerous conditions and primary gastric lymphoma of each case is presented as an estimated whole year. The diagnosis of gastric cancer or gastric precancerous conditions earlier than gastric lymphoma is indicated by a negative number, and the diagnosis of gastric cancer or gastric precancerous conditions later than gastric lymphoma is indicated by a positive number.

### 3.4. The risk of *H. pylori* infection in patients with primary gastric lymphoma, gastric cancer, or precancerous conditions

The *H. pylori* infection rate of primary gastric lymphoma patients was 68.4% (324/474), and as defined above the remaining patients were Hp-uninfected (20.0%, 95/474), and Hp-eradicated (11.6%, 55/474) (Table 5). Gastric antrum (*p* < 0.001) and pylorus (*p =* 0.014) involvement were more common in primary gastric lymphoma patients who were HP-infected, and patients with MALT lymphoma accounted for 56.2% of Hp-infected primary gastric lymphoma patients. MALT lymphoma was more common in Hp-infected primary gastric lymphoma patients compared to Hp-uninfected and Hp-eradicated patients (*p* < 0.001). The *H. pylori* infection rate of MALT lymphoma patients was significantly higher than that of DLBCL patients as well (MALT lymphoma, 182/216, 84.3% vs. DLBCL 123/225, 54.7%; *p* < 0.001). In addition, primary gastric lymphoma patients with Lugano classification stage I + II also had a higher *H. pylori* infection rate than patients with Lugano classification stage IIE + IV (*p* < 0.001). The *H. pylori* infection rate of primary gastric lymphoma with Lugano Classification stage I + II was as high as 89.5% (Table 5).

**Table 5.** *Helicobacter pylori* infection status of patients with primary gastric lymphoma, mean ± SD or *N*(%).

Characteristic	Primary gastric lymphoma (*n* = 474)			
	Hp-infected (*n* = 324)	Hp-eradicated (*n* = 55)	Hp-uninfected (*n* = 95)	*p* Value
Sex				0.808
Male	203 (62.7%)	35 (63.6%)	63 (66.3%)	
Female	121 (37.3%)	20 (36.4%)	32 (33.7%)	
Age, y	52.4 ± 5.9	54.3 ± 8.3	48.2 ± 6.8	0.327
Lesion location of lymphoma				
Gastric antrum	171 (52.8%)	23 (41.8%)	20 (21.1%)	< 0.001
Gastric body	181 (55.9%)	26 (47.3%)	58 (61.1%)	0.261
Gastric fundus	81 (25.0%)	11 (20.0%)	23 (24.2%)	0.726
Pylorus	82 (25.3%)	10 (30.9%)	11 (11.6%)	0.014
Cardia	26 (8.0%)	6 (10.9%)	10 (10.5%)	0.640
Lesion distribution of lymphoma				0.587
Multi-site	57(17.6%)	7 (12.7%)	14 (14.7%)	
Single-site	267(82.4%)	48 (87.3%)	81 (85.3%)	
Endoscopic manifestation of lymphoma				0.373
Ulcerative type	95 (28.4%)	19 (34.5%)	40 (42.1%)	
Mass or polypoid type	81 (25.0%)	14 (25.5%)	26 (27.4%)	
Fold change type	75 (23.1%)	13 (23.6%)	15 (15.8%)	
Infiltrative type	36 (11.1%)	5 (9.1%)	7 (7.8%)	
Superficial type	37 (13.1%)	4 (7.3%)	7 (7.8%)	
Pathological type of lymphoma				< 0.001
DLBCL	123 (38.0%)	31 (56.4%)	71 (74.7%)	
MALT lymphoma	182 (56.2%)	17 (30.9%)	17 (17.9%)	
Others	19 (5.9%)	7 (12.7%)	7 (7.4%)	
Lugano classification of lymphoma				< 0.001
I + II	290 (89.5%)	29 (52.7%)	44 (46.3%)	
IIE + IV	34 (33.1%)	26 (47.3%)	51 (53.7%)	
B symptoms of lymphoma				0.050
Yes	105 (32.4%)	10 (18.2%)	23 (24.2%)	
No	219 (67.6%)	45 (81.8%)	72 (75.8%)	
Complicated with gastric cancer				0.011
Yes	23 (7.1%)	1 (1.8%)	0 (0.0%)	
No	301 (92.9%)	54 (98.2%)	95 (100.0%)	
Complicated with gastric cancer of the intestinal type				0.040
Yes	14 (4.3%)	0 (0.0%)	0 (0.0%)	
No	310 (95.7%)	55 (100.0%)	95 (100.0%)	
Complicated with gastric cancer or precancerous conditions				< 0.001
Gastric cancer	23 (7.1%)	1 (2.8%)	0 (0.0%)	
Gastric precancerous conditions	63 (19.4%)	4 (7.3%)	2 (2.1%)	
None	238 (73.5%)	50 (90.9%)	93 (97.9%)	

DLBCL: diffuse large B-cell lymphoma; MALT; mucosa-associated lymphoid tissue; Hp: *Helicobacter pylori*.

For primary gastric lymphoma patients split into groups with gastric cancer, precancerous conditions, or neither, the *H. pylori* infection rate was significantly higher in the patients with gastric cancer and precancerous conditions (*p* < 0.001). Actually, gastric cancer (95.8%, 23/24) occurred mostly in Hp-infected primary gastric lymphoma patients and rarely occurred in patients after *H. pylori* eradication (4.2%, 1/24). None of our study subjects without *H. pylori* infection developed gastric cancer. Primary gastric lymphoma patients with a history of *H. pylori* infection (Hp-infected and Hp-eradicated) (with gastric cancer, 14/24, 58.3% vs. without gastric cancer, 146/450, 32.4%; *p* = 0.012) and present *H. pylori* infection (Hp-infected) (with gastric cancer, 23/24, 100.0% vs. without gastric cancer, 301/450, 66.9%; *p* = 0.003) had a higher risk of gastric cancer. The odds ratios for a history of *H. pylori* infection and present *H. pylori* infection were 1.068 (95% CI 1.040–1.096) and 1.069 (95% CI 1.035–1.105), respectively.

Similarly, gastric precancerous conditions also mostly occurred in Hp-infected primary gastric lymphoma patients (91.3%, 63/69), a few patients after *H. pylori* eradication (5.8%, 4/69), and rare Hp-uninfected patients (3.4%, 2/69). Additionally, gastric cancer of the intestinal type was more common in Hp-infected primary gastric lymphoma patients compared to Hp-uninfected and Hp-eradicated patients (*p* = 0.04) (Table 5). Gastric cancer of the intestinal type was only found in Hp-infected primary gastric lymphoma patients.

### 3.5. Treatment and prognosis of patients with primary gastric lymphoma, gastric cancer, or precancerous conditions

All Hp-infected primary gastric lymphoma patients received *H. pylori* eradication therapy, and the overall success rate of *H. pylori* eradication was 87.1%, with no difference in MALT lymphoma (89.9%), DLBCL (88.3%), and other pathological types (84.6%). *H. pylori* eradication therapy was effective in 40.9% of primary gastric lymphoma patients, 55.8% of MALT lymphomas patients, and 28.6% of DLBCL patients. Among them,17.7% of primary gastric lymphoma patients, 28.1% of MALT lymphoma patients, and 7.1% of DLBCL patients achieved complete remission. Only 2.9% of patients relapsed after complete remission with *H. pylori* eradication therapy. Finally, 7.4% of all primary gastric lymphoma patients achieved sustained complete remission after *H. pylori* eradication and didn’t need other treatments during follow-up, Most of which were MALT lymphoma patients (92.3%).

Primary gastric lymphoma patients with Lugano classification stage IIE + IV, and those who were Hp-uninfected, failed to eradicate *H. pylori*, or didn’t respond to *H. pylori* eradication therapy received chemotherapy (75.9%), targeted therapy (53.2%), surgery (12.4%), radiotherapy (6.8%), and immunotherapy (5.9%). Most patients (67.1%) received a combination of therapies. The prognosis of primary gastric lymphoma was generally well. The five-year overall survival rate and three-year overall survival rate of primary gastric lymphoma patients were 62.6% and 73.1%, respectively.

Among patients with primary gastric lymphoma and gastric precancerous conditions, 90.9% (10/11) of patients with low-grade intraepithelial neoplasia had atrophy before progressing, and 63.6% (7/11) had intestinal metaplasia during follow-up. All patients with intestinal metaplasia had atrophy either before or at the same time. While among patients with primary gastric lymphoma and gastric cancer, 91.7% (22/24) had atrophy, 66.7% (16/24) had intestinal metaplasia, and 16.7% (4/24) had low-grade intraepithelial neoplasia, either before or at the time of gastric cancer diagnosis. We found no statistical difference in the risk of developing metachronous gastric cancer between patients with primary gastric lymphoma who received and those who did not receive chemotherapy, as well as immunotherapy, radiotherapy, and targeted therapy.

Patients with primary gastric lymphoma complicated with synchronous gastric cancer and metachronous gastric cancer received surgery (70.8%), chemotherapy (37.5%), targeted therapy (25.0%), immunotherapy (16.7%), radiotherapy (8.3%), endoscopic submucosal dissection (16.7%), *H. pylori* eradication (75.0%), etc. The five-year overall survival rate and three-year overall survival rate of gastric adenocarcinoma in primary gastric lymphoma patients were 40.0% (6/15) and 57.9% (11/19), respectively, which were similar in patients with synchronous gastric cancer and metachronous gastric cancer. We found no significant difference in five-year overall survival rate (with gastric cancer, 6/15, 40% vs. without gastric cancer, 98/167, 58.7%; *p* = 0.161) and three-year overall survival (with gastric cancer, 12/19, 63.2% vs. without gastric cancer, 249/338, 73.7%; *p* = 0.315) between primary gastric lymphoma patients complicated with gastric cancer or not. The three-year overall survival rate (early gastric cancer, 5/5, 100.0% vs. advanced gastric cancer, 6/14, 42.9%; *p* = 0.045) and the five-year survival rate (early gastric cancer, 3/3, 100.0% vs. advanced gastric cancer, 3/12, 25.0%; *p* = 0.044) of early gastric cancer was significantly higher than that of the advanced gastric cancer in primary gastric lymphoma patients.

## 4. Discussion

We found a high risk of gastric cancer and precancerous conditions for primary gastric lymphoma patients from our multi-centre retrospective cohort study. A total of 5.1% and 14.6% of primary gastric lymphoma patients had gastric cancer and precancerous conditions, respectively. This is of particular concern in China, where the number of new cases and deaths of gastric cancer each year is close to 50% of all the world’s cases [22,23]. Chronic inflammation and the progressing cascade of precancerous conditions caused by *H. pylori* are high-risk factors for gastric cancer [24], and primary gastric lymphoma is a gastric malignant tumour second only to gastric adenocarcinoma in prevalence [3]. Pathological examination of gastroscopic biopsy is the gold standard for the diagnosis of primary gastric lymphoma. The majority of primary gastric lymphoma are of B-cell lineage pathologically, of which DLBCL accounts for 45% to 59%, and MALT lymphoma accounts for 38% to 48% of primary gastric lymphoma cases worldwide [25,26].

Cases of primary gastric lymphoma that are complicated with gastric adenocarcinoma are relatively rare in clinical practice, and therefore there are few studies on this topic, most of which are case reports and small-sample or single-centre studies. However, these previous studies and our research have shown that the presence of gastric malignant lymphoma increases the incidence of gastric cancer. Previous studies have shown that patients with primary gastric lymphoma face a significantly higher risk of developing other malignant tumours, including gastric cancer [27,28]. Amiot et al. [29] showed that patients with gastric MALT lymphoma had a 16-fold increased risk of gastric cancer compared with the French general population [29]. Our research also found that 5.1% of primary gastric lymphoma patients had gastric cancer, much higher than for the general Chinese population with a 5-year prevalence of 0.0276%. Inaba et al. [9] found that 7.2% (10/139) of Japanese patients with primary gastric lymphoma treated with radiotherapy developed metachronous gastric adenocarcinoma, which was higher than the prevalence of gastric adenocarcinoma in the general Japanese population. Old age, *H. pylori* infection, gastric mucosal change of chronic gastritis, and intestinal metaplasia were also found to be possible risk factors for metachronous gastric adenocarcinoma [9]. Capelle et al. [10] also showed that Dutch patients with gastric MALT lymphoma were six times (2.4%, 34/1419) more likely to have metachronous gastric adenocarcinoma than the general Dutch population, and Ishihama et al. [28] reported that 3.3% (4/121) of primary gastric malignant lymphoma patients develop synchronous gastric adenocarcinoma in Japan, a much higher figure than for the Japanese population in general (about 0.05%). The diagnosis interval between metachronous gastric cancer and primary gastric lymphoma varied widely. As previously reported, 91.2% to 100% of gastric cancer in primary gastric lymphoma patients were metachronous gastric adenocarcinoma developed after lymphoma [9,10]. Inaba et al. [9] reported that the mean latent period between primary gastric lymphoma and metachronous gastric adenocarcinoma was 43.1 months (range: 7.9 to 90.8 months). Our research reported 1.9% and 3.2% of primary gastric lymphoma patients had synchronous gastric cancer and metachronous gastric cancer, respectively, which were similar to those reported in previous literature, and higher than that in the general Chinese population.

There are even fewer studies on primary gastric lymphoma complicated with gastric precancerous conditions. However, the studies that have been conducted have found that atrophy and intestinal metaplasia appear in 54% to 91% and 51% to 70% of the surrounding tissue of gastric MALT, respectively [12,30]. In addition, Capelle et al. [10] found that 31% (440/1419) of Dutch gastric MALT patients had gastric precancerous conditions, with 4.6% having atrophic gastritis, 21.3% having intestinal metaplasia, and 5.1% having dysplasia. Later, they also found that precancerous conditions existed in 67.5% (27/40) of gastric MALT patients simultaneously and that the prevalence of atrophy, intestinal metaplasia, and dysplasia was 20%, 35%, and 12.5%, respectively [11]. No investigations of primary gastric lymphoma patients complicated with gastric cancer and precancerous conditions in China have previously been conducted.

Despite this, our observations for the prevalence of gastric cancer in primary gastric lymphoma patients are consistent with most earlier studies. However, we must pay attention to the endoscopic diagnosis of gastric adenocarcinoma both for the initial endoscopy and the endoscopic review during the follow-up of patients with primary gastric lymphoma. The prevalence of gastric precancerous conditions in primary gastric lymphoma is lower in our study than in earlier ones, and possible reasons for this include a lack of high-definition endoscopy, and insufficient knowledge and limited diagnostic ability of endoscopists on gastric precancerous conditions in the earlier years. Moreover, the sample size and time span of the study subjects are also factors that cannot be ignored. Insufficient amounts taken for biopsy might lead to insufficient bases for pathological diagnosis. In addition, the follow-up of gastric precancerous conditions in our study was consistent with Correa’s cascade [24].

This study also analyzed the clinicopathological characteristics of primary gastric lymphoma, gastric cancer, and precancerous conditions, including *H. pylori* infection. We found that most primary gastric lymphomas were ulcerative type and Lugano Classification stage I + II, and were mostly confirmed to be DLBCL and MALT lymphomas, and we also found that primary gastric lymphoma patients with ulcerative type and Lugano classification stage IIE + IV had a higher risk of developing gastric cancer and precancerous conditions as well. Furthermore, the *H. pylori* infection rate was higher in patients with MALT lymphoma, Lugano classification stage I + II, and patients with precancerous conditions and gastric cancer, especially gastric cancer of the intestinal type. Our findings on clinicopathological features of primary gastric lymphoma are consistent with those of other studies, which we discuss below.

One earlier study found that about two-thirds of primary gastric MALT lymphoma patients were diagnosed at stage I, and that stage IV accounted for only 4.2% [31]. Primary gastric lymphoma with stage IV had disseminated extranodal involvement or concomitant supradiaphragmatic nodal involvement according to the modified Lugano staging of primary gastrointestinal lymphoma [32]. We also had less than 10% of stage IV patients in our study. The ulcerative type was the most frequent presentation at endoscopy [31]. The appearance of an ulcer generally indicates invasion of the muscularis mucosae and deeper layers, which is related to severe Lugano classification stage and high-grade lymphoma [31]. It has also been shown that high-grade lymphomas presented more commonly as ulcerative type, being more frequently diagnosed in stage > I when compared with low-grade lymphomas [33]. We also found that the ulcerative type was an important risk factor for developing gastric cancer and precancerous conditions in primary gastric lymphoma, as was having a severe Lugano classification stage. A possible reason for this is that patients with ulcerative types and severe stages may have had a longer course of the underlying disease. For patients under follow-up care for primary gastric lymphoma, we need to distinguish metachronous gastric cancer and precancerous conditions from the recurrence of gastric lymphoma. We noticed that the median largest tumour size of 4 cm in metachronous gastric cancer was too large considering the median diagnosis interval of 3 years between primary gastric lymphoma and metachronous gastric cancer. A study of minute gastric cancer also showed that the mean growth rate was 0.0071 mm/day, and it took an average of 3.42 years to grow to 5 mm, with 95% of cases being well-differentiated tubular adenocarcinoma [34]. One possible reason for the larger tumour size of the metachronous gastric cancers in our study is that some gastric cancers may have been present before or at the time of diagnosis of primary gastric lymphoma but were missed. The small sample size of 15 metachronous gastric cancer patients is also a very important factor. In addition, 20% of metachronous gastric cancers were undifferentiated adenocarcinomas, which grow fast. All of these factors may exist, but no matter what the cause, gastric cancer should not be missed. Therefore, careful endoscopic and pathological evaluation is required to make the diagnosis.

Rentien et al. [35] found that precancerous conditions were more frequent in gastric MALT lymphoma than DLBCL and *H. pylori*-associated gastritis, and more frequent in the MALT lymphoma area than other areas in the stomach. However, our study didn’t find differences in the prevalence of precancerous conditions in gastric MALT lymphoma and DLBCL. Therefore, the prevalences of precancerous conditions among different pathological types of gastric lymphoma need to be further studied with large samples.

For our observed high *H. pylori* infection rate of primary gastric lymphoma, studies have even shown that *H. pylori* infection is related to adenocarcinoma of the distal stomach and gastric lymphoma [5,6,8]. Some studies have even shown that *H. pylori* infection was present in nearly 90% of primary gastric lymphoma patients [12,31]. *H. pylori* can cause chronic inflammation, gastric precancerous conditions, and intestinal-type gastric adenocarcinoma [6,8]. Therefore, we indeed observed that the *H. pylori* infection rate was higher in patients with primary gastric lymphoma complicated with gastric cancer and with precancerous conditions, and the underlying biological relationship between H. pylori, primary gastric lymphoma and gastric cancer, and precancerous conditions warrants further study. We also found that *H. pylori* eradication was effective in 40.9% of patients with primary gastric lymphoma, including 55% in MALT lymphoma and 28% in DLBCL, suggesting the necessity of eradicating *H. pylori* therapy in patients with primary gastric lymphoma. *H. pylori* eradication is the first choice for the treatment of gastric MALT lymphoma, and it has been reported so far that eradication of *H. pylori* can achieve a complete pathological response in 47% to 100% of *H. pylori*-positive gastric MALT lymphoma [5]. However, the role of *H. pylori* eradication in gastric DLBCL is still controversial. Gastric DLBCL with evidence of MALT is classified as DLBCL (MALT), which is not high-grade transformed MALT lymphoma or de novo DLBCL [5]. Several studies have found that some gastric DLBCL patients respond to *H. pylori* eradication therapy. A multicenter prospective study has reported that 80% of patients with early-stage gastric DLBCL (MALT) achieve complete remission at a median of 4.0 months after *H. pylori* eradication [36]. Several other studies also showed that some *H. pylori*-positive gastric DLBCL(MALT) patients respond to *H. pylori* eradication [37–39]. There were also cases of gastric de novo DLBCL patients who regressed after *H. pylori* eradication [40,41]. A study in Japan showed 27% of gastric de novo DLBCL patients achieved complete remission with eradication [42]. The efficacy of *H. pylori* eradication in DLBCL patients might be related to histological type and depth of invasion [42]. BCL10, NF-κB (p65), and CagA expression were also found to help predict the efficacy of *H. pylori* eradication in patients with early-stage gastric DLBCL(MALT) [36]. The results of our study and previous studies suggest that prospective studies on the efficacy of *H. pylori* eradication in gastric DLBCL patients are promising.

In addition, a previous study suggested an increased risk of metachronous gastric cancer in gastric DLBCL patients treated with chemotherapy, which can be explained by the fact that patients with advanced stages needed chemotherapy [9]. Amiot et al. [29] also found an increased risk of metachronous gastric cancer in gastric MALT Lymphoma patients who received immunotherapy. Our study didn’t find the negative effects of chemotherapy, radiotherapy, immunotherapy, and targeted therapy on the development of metachronous gastric cancer in primary gastric lymphoma patients. The association and mechanism of various therapies for lymphoma and the risk of solid tumours, including gastric cancer, remain to be further investigated. Furthermore, whether gastric cancer or gastric malignant lymphoma was treated first depended on the time of diagnosis. For patients with synchronous gastric cancer, the pathological types, stages and expected prognosis of gastric cancer and lymphoma should be taken into consideration to determine the treatment plan. Extending survival and improving quality of life is the primary goal at all times.

It’s also worth noting that we found no significant difference in prognosis between primary gastric lymphoma patients and complicated with gastric cancer. The main possible reason that gastric cancer didn’t significantly affect survival in primary gastric lymphoma patients was that the sample size was too small to represent the population. A previous study reported that the 5-year overall survival rates for gastric DLBCL and MALT lymphomas were 89.6% and 97.7%, respectively, while the 5-year survival rates for patients complicated with gastric cancer were 75.0% [10]. Differences in survival were not statistically analyzed in the study because only 139 patients with primary gastric lymphoma were included, 10 of whom had gastric cancer [10]. A few other studies had similar limitations. In addition, early gastric cancer accounted for 25% of patients with gastric cancer in our study, with a high survival rate and little impact on the prognosis. Regular follow-up of gastric lymphomas provided opportunities for the detection of early gastric cancer, which is also related to the detection and monitoring of precancerous conditions. One earlier study showed that up to 76% of gastric cancer in primary gastric lymphoma patients were of early type [28]. Other studies have shown increased prevalences and progression of intestinal metaplasia and dysplasia during follow-up for gastric lymphoma, which associated with the occurrence of carcinoma [32,43]. We also found a higher detection rate of early gastric cancer (25.0%) in primary gastric lymphoma patients than in the general Chinese population (11%) [44], which may explain the better prognosis and higher 5-year survival rate of gastric cancer complicated in primary gastric lymphoma patients (40.0%) than the gastric cancer patients (27.4%) in China [23]. Our study also found that the development of gastric cancer and precancerous conditions complicated in primary gastric lymphoma conformed to Correa’s cascade [24]. Therefore, the follow-up of patients with primary gastric lymphoma may provide an opportunity for early detection of gastric cancer, thereby improving the prognosis of gastric cancer.

The primary advantage of our study is that it is the first Chinese study of primary gastric lymphoma complicated with gastric cancer and precancerous conditions. Moreover, our study is a multi-centre study and has the largest sample size among the current studies on gastric lymphoma complicated with gastric cancer and precancerous conditions, followed by the Dutch study [10]. There are also some limitations to the study. The three centres included in this study are located in urban areas in China, two of which are located in the same city. Therefore, there may be bias among the study subjects. In addition, endoscopists could diagnose atrophic gastritis and intestinal metaplasia based on endoscopic findings, and biopsy pathology was not performed on all these patients. Although pathology is the ‘gold standard’, pathological evaluation of gastric atrophy and intestinal metaplasia is also based on endoscopic observation by endoscopists and is related to the sampling location and number. Endoscopic evaluation of gastric atrophy and intestinal metaplasia also relies on the endoscopic equipment and the experience of the endoscopists. Conventional white-light endoscopy showed a poor correlation between histological and endoscopic findings in the diagnosis of gastric atrophy and intestinal metaplasia [16]. High-definition white-light endoscopy can identify atrophy and intestinal metaplasia better than conventional white-light endoscopy [45]. Narrow-band imaging and white-light endoscopy nontargeted biopsies may present more promising results for the diagnosis of atrophy and intestinal metaplasia [46,47]. Narrow-band imaging-guided biopsies further improved the diagnostic yield of atrophy and intestinal metaplasia [48]. We included patients with atrophy and intestinal metaplasia both endoscopically and pathologically diagnosed in the gastric precancerous conditions group. While high definition white-light endoscopy and/or narrow-band imaging were used in most cases, except in some early cases. Furthermore, because of our lack of knowledge about the grading of atrophy and intestinal metaplasia, many patients were not graded, especially before 5 years ago. We cannot accurately judge the severity of atrophy or intestinal metaplasia according to the endoscopic pictures at present. However, if we discard cases that are not graded for precancerous conditions, we will lose a lot of cases and information, and we will underestimate the probability of precancerous conditions in patients with primary gastric lymphoma. Therefore, we included these patients with precancerous conditions that were not graded for severity in the precancerous conditions group. It does cause bias.

## 5. Conclusion

In summary, we find that patients with primary gastric lymphoma have a high risk of developing gastric cancer and precancerous conditions and that this risk may be related to *H. pylori* infection. Follow-up of primary gastric lymphoma may provide an opportunity for early detection of gastric cancer. Endoscopists should pay attention to the endoscopic findings of metachronous gastric cancer, synchronous gastric adenocarcinoma and precancerous conditions in patients with primary gastric lymphoma.

## Data Availability

The data used to support the findings of this study are available from the corresponding author upon request.
